# Effects of Pre-Processing Hot-Water Treatment on Aroma Relevant VOCs of Fresh-Cut Apple Slices Stored in Sugar Syrup

**DOI:** 10.3390/foods9010078

**Published:** 2020-01-10

**Authors:** Guido Rux, Efecan Efe, Christian Ulrichs, Susanne Huyskens-Keil, Karin Hassenberg, Werner B. Herppich

**Affiliations:** 1 Department of Horticultural Engineering, Leibniz Institute for Agricultural Engineering and Bioeconomy (ATB), 14469 Potsdam, Germany; efeefeca@cms.hu-berlin.de (E.E.); khassenberg@atb-potsdam.de (K.H.); wherppich@atb-potsdam.de (W.B.H.); 2 Research Group Quality Dynamics/Postharvest Physiology, Division Urban Plant Ecophysiology, Humboldt-Universität zu Berlin, 14195 Berlin, Germany; christian.ulrichs@hu-berlin.de (C.U.); susanne.huyskens@hu-berlin.de (S.H.-K.)

**Keywords:** minimal processing, sugar syrup immersion, volatile organic compounds, chemical prevention, ready-to-eat fruit salads

## Abstract

In practice, fresh-cut fruit and fruit salads are currently stored submerged in sugar syrup (approx. 20%) to prevent browning, to slow down physiological processes and to extend shelf life. To minimize browning and microbial spoilage, slices may also be dipped in a citric acid/ascorbic acid solution for 5 min before storage in sugar syrup. To prevent the use of chemicals in organic production, short-term (30 s) hot-water treatment (sHWT) may be an alternative for gentle sanitation. Currently, profound knowledge on the impact of both sugar solution and sHWT on aroma and physiological properties of immersed fresh-cuts is lacking. Aroma is a very important aspect of fruit quality and generated by a great variety of volatile organic compounds (VOCs). Thus, potential interactive effects of sHWT and sugar syrup storage on quality of fresh-cut apple slices were evaluated, focusing on processing-induced changes in VOCs profiles. Intact ’Braeburn’ apples were sHW-treated at 55 °C and 65 °C for 30 s, sliced, partially treated with a commercial ascorbic/citric acid solution and slices stored in sugar syrup at 4 °C up to 13 days. Volatile emission, respiration and ethylene release were measured on storage days 5, 10 and 13. The impact of sHWT on VOCs was low while immersion and storage in sugar syrup had a much higher influence on aroma. sHWT did not negatively affect aroma quality of products and may replace acid dipping.

## 1. Introduction

Fresh-cut processing induces a catena of physiological responses [1,2] finally resulting in the loss of quality and aroma and pronouncedly shortens the storage life of fresh-cut produce [3]. In current practice, fresh-cut fruit for fruit salads are often stored in sugar syrup (ca. 20%), especially for use by bulk purchasers [4]. This storage method may extend product shelf life by preventing enzymatic and oxidative browning and transpiration, slows down respiration, ethylene metabolism and other physiological processes [5,6,7]. In this context, however, microbial spoilage is the main factor limiting shelf life [7]. It is therefore very important to remove the microorganisms adherent to the fruit skin [8] before processing.

In practice, e.g., apple slices are dipped in a mixture of citric and ascorbic acid solutions for sanitation purposes. To prevent the consumption of these chemicals especially in organic production, gentle physical sanitation methods are demanded.

As such, hot-water treatments (HWT) in the temperature range of 40–80 °C were shown to effectively reduce microbial contamination, and are relatively inexpensive and easy to use [9]. In addition, HWT maintains storage quality of fruits [10,11,12]. Since they are chemical-free, particularly short-term (15–60 s) hot-water treatments (sHWT) are suitable for organic production [13,14]. Besides earlier studies on the impacts of HWT on structure and function of fruit epidermal tissue [15,16], only recently, the effects of sHWT on surface tissue, heat transfer dynamics and suitability for pre-processing of intact apples for fresh-cut salads were investigated in detail [12,17]. Furthermore, the implications of sHWT on important quality parameters such as tissue browning, tissue strength and on microbial loads of apple slices immersed in sugar syrup have been studied under semi-practical conditions [18]. On the effects of sugar syrup storage on fresh-cut fruit quality attributes only very few studies are available [7,19].

In contrast to analyses of sHWT impacts on visual and internal quality attributes of fresh-cut fruit [12,17,18], studies on potential variations in fruit aroma, a very important aspect of fruit quality sensation [2,20], are completely lacking. Aroma is generated by a great variety of permanent or secondary VOCs [21], synthesized via numerous biosynthetic pathways, which, in turn, are regulated by a great variety of enzymes and substrates [2,22,23]. Moreover, both respiration and ethylene biosynthesis are also involved in VOCs evolution [24]. In addition, microbial growth may, directly or indirectly, negatively affect product aroma [25,26]. Short-term-HWT and sugar syrup immersions may influence all of these processes. Knowledge on the impact of both sugar solution and sHWT on aroma development and physiological properties of immersed fresh-cuts is lacking.

Thus, the present study focused on the evaluation of the potential effects of sHWT (30 s, at 55 °C or 65 °C) on the aroma-related quality of ‘Braeburn’ apple slices stored in sugar syrup under strictly simulated practical condition. Some parts of the samples were also pre-treated with a commercial ascorbic/citric acid solution to test the potential synergistic effects of this treatment on aroma. During a 13 days-storage at 4 °C, respiration, ethylene emission and, for the first time, the processing-induced direct and indirect changes in VOC profiles were measured at defined intervals. This will enable comprehension of the respective quality-related physiological processes, to effectively select the optimal process conditions and to verify whether sHWT can safely replace the use of chemicals in processing of ecologically produced fresh-cut fruit salads.

## 2. Materials and Methods

### 2.1. Material

Fresh mature ’Braeburn’ apples (*Malus domestica* Borkh.) were obtained from a commercial fresh-cut salad producer. At the Department of Horticultural Engineering (Leibniz Institute for Agricultural Engineering and Bioeconomy, Potsdam, Germany), the apples were stored at 4 °C and 95% relative humidity for up to 3 days until the start of the experiments. Undamaged apples of uniform size (mean fresh mass: 150.7 ± 5.1 g and mean dry matter content: 177 ± 11 g kg^−1^) were selected.

### 2.2. Pre-Processing Short-Term Hot-Water Treatment

Before cutting, apples were divided in five batches of 16 fruit each. Apples from the control batch were water-washed at approx. 20 °C. The other samples were hot-water-treated in a GFL 1086 water bath (Gesellschaft für Labortechnik mbH, Burgwedel, Germany) by submerging four apples each for 30 s. Samples of two batches each were hot-water-treated at 55 °C or 65 °C, respectively, according to Kabelitz and Hassenberg [12] and Kabelitz et al. [19]. These authors indicated that HWT at 55 °C for 30 s effectively reduced microbial loads without negatively affecting the external quality of samples, while 65 °C showed to be a negative control treatment in terms of quality maintenance.

### 2.3. Fresh-Cut Preparation and Sampling

**Figure 1:**
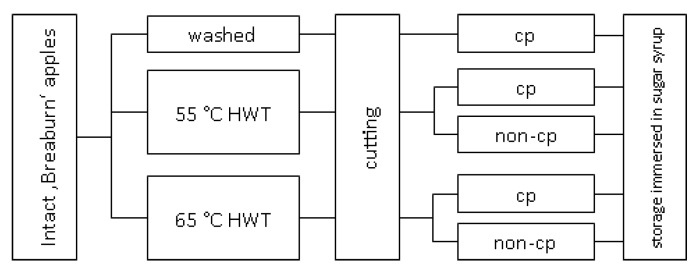
Scheme of the various pre- (short-term hot-water, sHWT, vs. washed) and post-cutting (chemical prevention, cp, vs. no chemical prevention, non-cp) treatments of fresh-cut apples slices stored in sugar syrup at 4 °C for up to 13 days.

After washing and hot-water treatments, all apples were cut under semi-practical hygienic conditions in a cooling room at 4 °C. For this, apples were at first halved equatorially and then each half-segment was cut into 16 pieces by a Parti apple cutter/corer (Gefu GmbH, Eslohe, Germany). The controls and slices of one of each 55 °C and 65 °C sHWT batches were additional chemically treated (= chemical prevention, cp) by immersing in ascorbic/citric acid solution (40 g ascorbic and 20 g citric acid solved in 1 L deionized water) for 5 min (Figure 1). Slices of each batch were randomly filled in 12 commercial 840 mL plastic pails. Closely following practice, each pail containing approx. 48 apple slices (approx. 228 g) was filled up with 450 mL sugar syrup (200 g L^−1^ invert sugar syrup, 72.7%; Hanseatische Zuckerraffinerie GmbH & Co. KG, Hamburg, Germany plus 10 g L^−1^ OBSTSERVAL HC-2 browning inhibitor (Konserval, Pharmacon Lebensmittelzusätze GmbH, Trittau, Germany). The browning inhibitor contained ascorbic acid, sodium ascorbate and citric acid. All pails were tightly closed and stored at 4 °C for up to 13 days. Three pails (replicates) of each batch were opened on days 5, 10 (the common maximum shelf-life) and on day 13. For further analyses, 26 apple slices of each pail were removed.

### 2.4. Sampling of Volatile Organic Compounds (VOCs) and Ethylene

To determine VOCs emission, ethylene evolution and respiration rate (RR), 20 apple slices were filled in a glass jar (1 L), which was hermetically closed by a glass lid. VOCs emitted by samples accumulated at 4 °C for 1 h. Then VOCs were 10 min-extracted with 50/30 μm divinylbenzene/carboxen/polydimethylsiloxane (DVB/CAR/PDMS) SPME fiber (stableFlex/SS, Supelco, Bellefonte, PA, USA) via septum in glass lid. After VOCs extraction, 0.5 mL sample air was taken from headspace with a 0.5 mL A-2 Series syringe (VICI Precision Sampling, Baton Rouge, LA, USA) via the same septum to measure ethylene and CO_2_ concentrations.

### 2.5. Analysis of Volatile Organic Compounds

VOCs were analyzed with a GCMS-QP2010 SE gas chromatograph-mass spectrometer (GC–MS; Shimadzu Europa GmbH, Duisburg, Germany) on a DB-WAX (Agilent Technologies, Palo Alto, CA, USA) column (30 m × 0.25 mm × 0.25 μm). SPME fiber was introduced into the GC injector (set to 250 °C and fitted with a SPME liner (0.75 mm × 5.0 mm × 95 mm); Shimadzu GCs; Restek, Bellefonte, PA, USA) for 30 min for VOC desorption and conditioning before next extraction. Helium was used as carrier gas (flow rate: 0.8 mL min^−1^). The following temperature program was used: 35 °C (hold 1 min), ramp to 110 °C at 5 °C min^−1^ and ramp to 230 °C at 10 °C min^−1^ (hold 3 min). The mass spectrometer (MS) detector worked in full scan mode (mass-to-charge ratio: 35–250 m/z) and operated in electron impact mode at 70 eV. Measured chromatogram peaks were integrated based on quantifier and qualifier ions of target compound (Table 1) using MSD Chem-Station E.02.01.1177 (Agilent Technologies, Palo Alto, CA, USA). Specific VOCs were identified (1) by NIST v2.0f library (NIST, Gaithersburg, MD, USA) and (2) by retention index (RI) calculated for each compound according to van Den Dool and Kratz [27]. RIs were determined based on an alkane standard (C7–C30; 1000 μg mL^−1^; Supelco, Bellefonte, PA, USA). For this, 5 μL of alkane standard was injected in a 3.9 L glass jar, extracted with SPME after equilibrium time of 1 d and analyzed as described above. The RIs were compared with those given by van Den Dool and Kratz [27] by using online NIST Chemistry WebBook [28]. The total integrated peak area of each VOC was used to calculate a semi-quantitative VOC concentration expressed in nL L^−1^ based on the external standard 2-methylbutyl 3-methylbutanoate (CAS-number: 2245-77-4). A standard curve of 2-methylbutyl-3-methylbutanoate was created in the concentration range from 5 to 1000 nL L^−1^.

### 2.6. Quantification of Ethylene and CO_2_ Evolution

A small volume of 0.5 mL air was taken from canning glass jar and analyzed with a GC17A gas chromatograph (Shimadzu) to measure ethylene and CO_2_ concentrations. The GC was equipped with both FID and TCD, 80/100 Porapak N (Supelco, Bellefonte, PA, USA) column (1.8 m × 1/8 in × 2.1 mm) and a 5605PC mole sieve (Alltech GmbH, Unterhaching, Germany). As carrier gas, helium was used (constant flow rate: 1.4 mL min^−1^), oven temperature was kept constant at 60 °C. Measured peaks were integrated using Class-VP chromatography data system software v4.2 (Shimadzu Europa GmbH, Duisburg, Germany). Rates of ethylene release were expressed in µg kg^−1^ h^−1^, those of CO_2_ production in mg kg^−1^ h^−1^.

### 2.7. Statistical Analysis

For each treatment, three pails were used as replicates for each sample day. VOCs and ethylene emissions were each determined on 20 apple slices from each pail (*n* = 3), while respiration was analyzed on a total of 60 apple slices, i.e., 20 from each pail. Statistical analyses (ANOVA) were carried out with WinSTAT (R. Fitch Software, Staufen, Germany) and results were presented as mean ± standard deviation (SD). The significance of differences between the calculated means was analyzed by Duncan’s multiple range test (*p* < 0.05). The relationships between VOC evolution and sHWT were additionally analyzed by principal components analysis (PCA) using Latentix Ver. 2.00 (Latent5, Copenhagen, Denmark).

## 3. Results

### 3.1. Volatile Organic Compounds

In total, 33 different VOCs were identified in fresh-cut apple slices, with esters (21) forming the major group of compounds (Table 1). The emission of individual VOCs was significantly affected by cutting, sHWT and/or storage time (Table 2). In fresh, intact ’Braeburn’ apples, 2-methylbutyl acetate (29.0%) and α-Farnesene (15.9%) were the most abundant VOCs. Cutting immediately and significantly increased the emissions of 17 VOCs, while that of only three VOCs significantly decreased. The cumulative VOCs concentration increased sevenfold, particularly concentrations of acetate esters (es2–es8) increased most.

During further storage, the emissions of the majority of VOCs were significantly reduced compared to those observed immediately after cutting. Cumulative VOCs concentrations decreased by 37.7% for control and by 18.8%–50.7% for sHWT samples, but were nevertheless 3.5–5.8 times higher than for intact apples (Table 2, 1st position). While cutting immediately and strongly increased the emission of acetate esters, the concentrations of these compounds decreased or, at least, remained constant during storage. Only the concentration of ethyl-acetate continued to increase after 3 days of storage as did the concentrations of all other ethyl esters, methyl 2-methylbutyrate, ethanol, hexanal and both ketones.

Responses of sHWT samples depended on both the treatment temperatures and the post-processing acid treatment (Table 3). Head space concentration of ethanol was generally higher and that of estragole and propyl-acetate lower in sHW-treated samples than in controls; propyl acetate concentration was more reduced at 65 °C than at 55 °C. Furthermore, sHWT at 65 °C significantly reduced emissions of ethyl-propionate, ethyl hexanoate and methyl hexanoate. These VOCs were also reduced at sHWT of 55 °C combined with acid treatment, i.e. chemical prevention. The combination of sHWT and acid application also resulted in increased D-Limonene and decreased ethyl 2-butenoate emissions, while the enhancement of the ethanol emission was attenuated. At a sHWT of 55 °C, the combination with organic acid resulted in lower ethyl acetate emission. In contrast, only the sole sHWT at 55 °C (i.e., without cp) let the emissions of nearly all ethyl esters increase especially that of ethyl-acetate, methyl hexanoate and 2-butanone, compared to controls.

**Figure 2:**
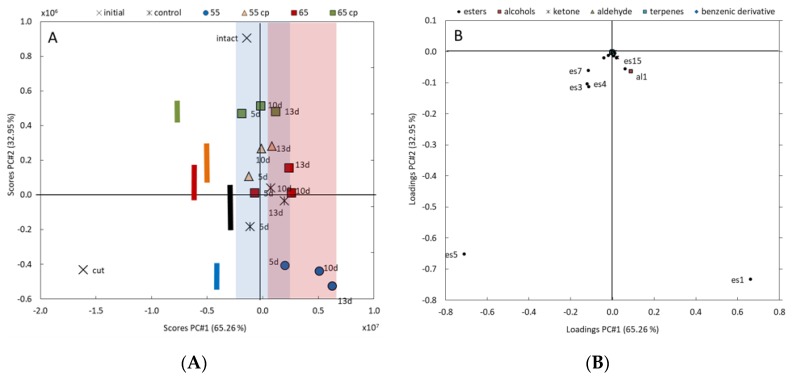
(**A**) Scatterplot of principal components analysis (PCA)-results of the variability of VOCs profiles during storage of treated and untreated fresh-cut apple slices. Decreasing scores of PC1 and PC2 can be associated with the effects of cutting; scores values of PC1 are associated with storage time, marked by colored areas (blue = 3 days and red = 10 days); decreasing scores of PC2 additional associated with distinct effects of the various treatments, marked by colored bars. (**B**) Loading plot of PCA-results of single-VOC impact on the total variance in VOCs profiles. VOCs with negative loadings of both PCs can be associated with the effects of cutting; VOCs with positive PC1 and negative PC2 loadings are related during storage and distinct effects of the treatments.

The PCA provides two principal components (PC), which together represent 98.2% of the total variance in VOCs profiles. The effects of cutting, sHWT temperature and duration of storage can be visualized by a scatter point plot (Figure 2A). Decreasing values of PC1 (representing 65.3% of total variance in VOCs profiles) can be associated with the effects of cutting, while increasing values are associated with storage time. Decreasing values of PC2 (representing 33.0% of total variance in VOCs profiles) can also be associated with cutting, and, beyond that, with the distinct effects of the various treatments. Interestingly, sHWT at 55 °C induced the highest alteration of the VOCs profile from that of intact apples (c.f. colored bars in Figure 2A).

For both PCs, the impact of each single VOC on the total variance in the VOCs profiles can be identified as visualized in Figure 2B. Mostly, ethyl acetate (es1) and, to a much smaller extent, ethyl 2-methylbutanoate (es15) and ethanol (al1) are related to effects of the sHWT (PC2), but also to changes during storage (PC1). Cutting-induced changes in the VOCs profiles (both PC decreased) are mainly associated with 2-methylbutyl acetate (es5), but also with isobutyl acetate (es4), butyl acetate (es3) and hexyl acetate (es7).

### 3.2. Ethylene Evolution

**Figure 3:**
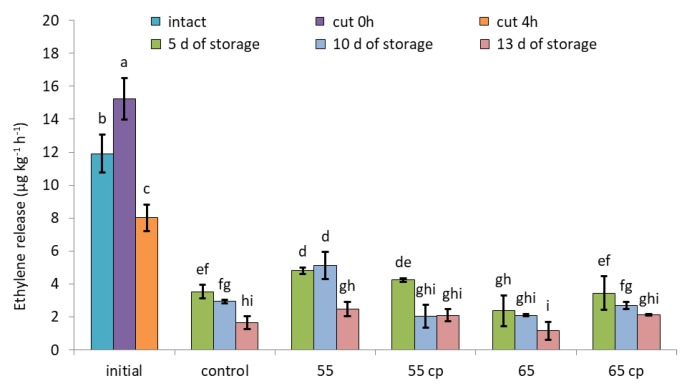
Ethylene release rates of short-term hot-water treated and untreated fresh-cut apple slices at days 5, 10 and 13 of storage in sugar syrup at 4 °C, compared to intact apples and untreated fresh-cut apple slices (initial). Given are means ± standard deviation (*n* = 3). Different letters indicate significant differences between means (*p* < 0.05). 5 d = 5 days, 10 d = 10 days, 13 d = 13 days.

Cutting immediately and significantly intensified ethylene release of untreated apple slices by approx. 30%; however, ethylene emission also rapidly, within 4 h, declined to rates below that of intact apples (Figure 3). Storage in sugar syrup generally reduced ethylene release as measured after removing the samples from the solution. Compared to controls, sHWT at 55 °C initially (i.e., at day 5 of storage) intensified ethylene emissions of apples slices, while that at 65 °C pronouncedly lowered them. Irrespective of treatments, ethylene emission of all apple slices continuously declined during further storage (Figure 3; days 10 and 13).

### 3.3. Respiration

**Figure 4:**
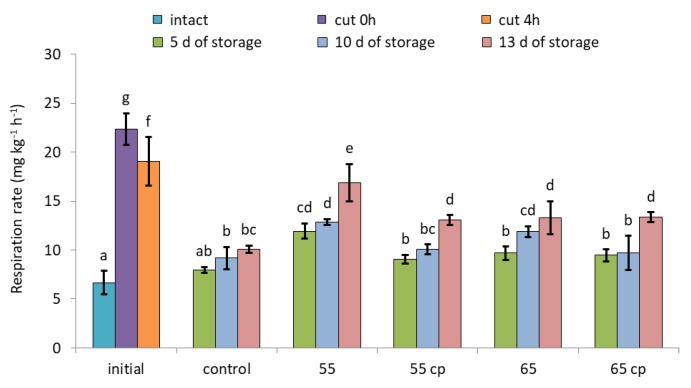
Respiration rates of short-term hot-water treated and untreated fresh-cut apple slices at days 5, 10 and 13 of storage in sugar syrup at 4 °C, compared to intact apples and untreated fresh-cut apple slices (initial). Given are means ± standard deviation (*n* = 3). Different letters indicate significant differences between means (*p* < 0.05). 5 d = 5 days, 10 d = 10 days, 13 d = 13 days.

CO_2_-based respiration rates of untreated ’Braeburn’ apples increased 3.4 times immediately after cutting and then slightly declined again within 4 h (Figure 4). During storage in sugar syrup, CO_2_ release of all samples was higher than that of intact apples. In addition, respiration of sHWT-samples treated at 55 °C without acid treatment was significantly higher than that of the other apple slices at all sampling days.

## 4. Discussion

In the presented experiment, several overlaying and potentially interactive effects were responsible for the development of respective VOCs profiles during storage of apple slices, i.e., cutting, sHW-treatment at two temperatures and storage in sugar syrup with its pronounced effect on O_2_ availability. The most pronouncedly emitted VOCs were esters. Similarly, Paillard [31] reported that esters of acetic, butanoic and hexanoic acids with ethyl, butyl and hexyl alcohols are the most frequent VOCs detected in the headspace of intact apples. The respective composition of the above esters determines the typical fruity (i.e., ’apple-like’) cultivar-specific aroma of apples [32]. In the presented study, 10 of the identified esters were described as generally important “character impact” VOCs [29]. Although the overall composition of the VOCs may clearly vary among different apple cultivars [33], 2-methylbutyl acetate and α-Farnesene are most often the major components in ’Braeburn’ apples [34,35]. This was confirmed by the present results.

### 4.1. Effect of Cutting on the Release of VOCs, Ethylene and CO_2_

The cutting-related increase in acetates probably impacted the aroma of fresh-cut apples, as 2-methylbutyl acetate, butyl-acetate and hexyl acetate are characterized as “character impact compounds” [29]. These VOCs are predominantly associated with a characteristic sweet, cox-like apple aroma [36]. The presented results confirmed earlier findings that cutting inevitably induces physiological wounding-stress responses characterized by temporarily increased ethylene synthesis [37,38], respiration activity [7] and VOCs release [39], which all return to pre-processing levels within 24 h [40]. Emissions of acetate-esters may strongly increase, due to increased lipoxygenase activity [41] resulting in enhanced esterification of membrane lipids [42,43]. Furthermore, a largely accelerated glycolysis increases the acetyl-CoA availability, and thus also boosts amino-acids production, which serve as precursors to many VOCs [43,44]. During storage, however, the concentrations of the aroma-relevant acetates decreased, which may indicate that the cutting induced stress response was reduced or no longer existed. Therefore, the generation of precursors relevant for VOC synthesis was reduced resulting in a decline of emissions. However, the emissions were still higher than those from intact apples. Nevertheless, the typical aroma, appearing immediately after cutting the apples, got continuously lost during storage. This, however, is generally monitored during storage of fresh-cut apples [44].

### 4.2. Impact of Hot-Water Treatment on the Release of VOCs, Ethylene and CO_2_

Even the sHWT at 65 °C did not alter the emission of most VOCs or pronouncedly impacted VOCs profiles but only marginally reduced that of few esters. In contrast to cutting, sHWT did obviously also not affect the synthesis of acetate-esters. This is surprising because a high number of VOCs emanate from the epidermal layers of apples, while only a lower proportion derived from the pulp tissue [45]. However, dipping intact apples for 30 s in hot-water at 55 °C exclusively heated the epidermis and few hypodermal cell layers [17]. Furthermore, although treatments at higher temperatures (i.e., >55 °C) resulted in faster increase in tissue temperature of deeper cell layers, only 70 °C pronouncedly damaged the epidermis [17]. Thus, sHWT, in the temperature range used, did not decrease the emission of important “character impact” compounds [29] or increase that of VOCs associated with off-odor. Therefore, sHWT did not reduce the aroma quality. Similarly, sHWT (of up to 65 °C) did not adversely affect color attributes, tissue strength or other important quality parameters of fresh-cut apple slices [18].

Although the increased ethanol emission may be assumed as critically, the aroma-relevant threshold for this compound is much higher than for other important VOCs (Table 3). In addition, the ethanol concentrations in total were very low. The observed increase in ethanol emission, therefore, did not negatively affect the aroma quality at all. It may, nevertheless, indicate some sHWT-induced heat stress because ethanol and ethyl-acetate emissions are known to increase in response to and may be used as indicators of this stressor [46,47]. Heat stress was reported to alter the glycolytic pathway by disturbing the mitochondrial electron transport, which, similar to reduced O_2_ availability, results in increased ethanol formation [48]. This may also be reflected by the marginally higher respiratory activity observed at the end of storage. Heat treatment-enhanced respiration was also reported earlier for intact ‘Granny Smith’ and ‘Anna’ apples [49]. In addition, the increased D-limonene emissions of acid-treated sHWT samples may be attributed to major perturbations of the cellular metabolism that result in the expression of multiple genes and, finally, in enhanced terpene emissions [50,51].

Omitting the post-processing acid treatment increased the ethanol emission, which, however, did not negatively affect the aroma (see above). Hot-water treatments without post-processing acid application only slightly enhanced the alteration of the VOCs profile typical for intact apples in comparison to those with additional acid dipping. Interestingly, sHWT at 55 °C without additional acid treatment clearly intensified the storage-induced physiological responses of fresh-cut apple slices, i.e., it slightly enhanced their respiration activity and ethylene release. This may indicate a direct response of the treated apples to moderate but not to excessive heat or to acid pretreatment [52,53]. Additionally, the emission of ethyl esters is further intensified in sHW-treated (55 °C) apple slices. This is especially obvious for ethyl acetate and ethanol, which, however, both increased in all samples during storage (see Section 4.3). As ethylene may directly regulate the synthesis of VOCs [44,54,55], the increase in ethyl 2-methylpropanoate, ethyl butyrate and ethyl 2-methylbutanoate may be directly related to the enhanced ethylene tissue concentrations. Ethyl acetate correlates with pronounced off-odor [56], while the “character impact compounds” [29] ethyl-butyrate and ethyl 2-methylbutanoate are generally associated with fruity and apple-like aroma [57]. The increase in the head space concentrations of the latter compounds, which own aroma thresholds much lower than ethyl acetate [29,30], may thus even improve the overall aroma of the apple samples (Table 3).

### 4.3. Effects of Storage in Sugar Syrup on the Release of VOCs, Ethylene and CO_2_

Storing fresh-cut apple slices in sugar syrup pronouncedly affected the development of their VOCs profiles, as indicated by the distinct increase in ethyl ester emissions. Following sHWT, head space concentration of ethyl acetate of samples increased 0.5–1.6-fold compared to controls; it, however, increased approx. 100 times due to the sugar syrup.

The reduction of ethylene and CO_2_ release observed after immersion in the syrup, corroborate the findings of Rux et al. [7]. These authors stored apple slices in sugar syrups of different concentrations and attributed major changes in respiration of samples to the reduced O_2_ availability due to impaired gas exchange between tissues and ambient air [7,58]. Therefore, the fast decrease in O_2_ and increase in CO_2_ concentrations within the tissue increasingly inhibits physiological processes [59]. The increased emissions of ethanol and other alcohols probably indicate semi-anaerobic conditions [60].

Likewise, the increased emissions of ethyl esters may be explained by the increased ethanol formation under these conditions. Alcohol acetyl-CoA transferase (AAT) catalyzes the esterification of carboxylic acids and alcohols [46,61], and enhanced ethanol concentrations may enhance the formation of ethyl esters, especially ethyl acetate [62]. In this context, Cortellino et al. [62] measured a progressive increase of ethyl acetate in fresh-cut apple tissue within 11 days of storage in modified atmosphere, most pronounced at an O_2_ concentration of 1%.

However, in this study, the influence of oxygen availability on VOC synthesis during storage in sugar syrup submerged apple pieces cannot be sufficiently clarified. Further studies focusing on oxygen availability are necessary.

## 5. Conclusions

In a semi-practical approach, this study comprehensively evaluated the potential food quality-related effects of sHWT (at 55 °C and 65 °C), cutting and/or post-processing acid treatment and their interactions on the development of aroma-relevant VOCs as well as on the physiological indicators ethylene synthesis and respiration activity in fresh-cut apple slices stored in sugar syrup. Cutting temporarily enhanced ethylene synthesis, respiration activity and VOCs production. In this context, 2-methylbutyl acetate, butyl acetate and hexyl acetate were identified as most relevant cutting-enhanced VOCs, but their emissions declined again during syrup-storage. Syrup-storage increased ethanol and ethyl-esters, and in particular ethyl-acetate, which might be attributed to reduced O_2_ availability under this condition. Solely applying sHWT at both 55 °C and 65 °C enhanced the respiration of apples slices, which potentially indicated marginal heat stress. This, however, only slightly intensified ethylene release but increased ethanol emission of samples, indicating alterations in respiratory pathways. Additionally, sHWT at 65 °C resulted only in minor declines of few esters. Nevertheless, the general impact of sHWT on VOCs profiles of apples slices was low, especially in comparison to storage in sugar syrup and did not negatively affect aroma. The omission of pre-processing acid application did not negatively affect the aroma to a crucial degree. Consequently, sHWT might replace the post-processing acid treatments for fresh-cut products.

## Figures and Tables

**Table 1:** All identified volatile organic compounds (VOCs) emitted by intact, freshly cut and variously treated (sHWT/chemical prevention) fresh-cut apple slices as characterized by their CAS registry numbers, calculated retention indices (RI), previously reported retention indexes (RI_literature_), and quantifier and qualifier ions used for peak integration.

Chemical Group	VOCs	CAS-Number	RI	RI_literature_	Quantifier (Qualifier) Ions (m z^−1^)
esters (21)	Ethyl acetate	141-78-6	903	863–908	43 (45–61)
	Ethyl propionate	105-37-3	963	939–976	57 (74–75)
	Ethyl 2-methylpropanoate	97-62-1	968	957–969	43 (41–71)
	Propyl acetate	109-60-4	977	952–996	43 (61–73)
	Methyl butyrate	623-42-7	988	969–993	74 (43–71)
	Methyl 2-methylbutyrate	868-57-5	1011	1000–1010	57 (41–88)
	Isobutyl acetate	110-19-0	1014	1000–1031	43 (56–73)
	Ethyl butyrate	105-54-4	1035	1000–1073	71 (43–88)
	Ethyl 2-methylbutanoate	7452-79-1	1050	1022–1073	57 (41–102)
	Butyl acetate	123-86-4	1069	1049–1105	43 (56–73)
	2-methylbutyl acetate	624-41-9	1116	1111–1125	43 (55–70)
	Ethyl valerate	539-82-2	1129	1131–1139	88 (57–85)
	Ethyl 2-butenoate	10544-63-5	1154	1158–1158	69 (41–99)
	Pentyl acetate	628-63-7	1165	1175–1181	43 (55–70)
	Methyl hexanoate	106-70-7	1178	1176–1189	74 (43–99)
	Ethyl hexanoate	123-66-0	1222	1196–1245	71 (43–89)
	Hexyl acetate	142-92-7	1258	1251–1311	43 (56–61)
	2-Hexen-1-yl acetate	10094-40-3	1317	-	
	Hexyl butyrate	2639-63-6	1397	1393–1410	71 (43–89)
	Hexyl 2-methylbutanoate	10032-15-2	1414	1415–1416	
	Hexyl hexanoate	6378-65-0	1610	1596–1599	43 (56–117)
ketones (2)	2-Butanone	78-93-3	908	875–926	43 (57–72)
	1-Penten-3-one	1629-58-9	1020	1019–1056	
alcohols (6)	Ethanol	64-17-5	943	900–955	45 (43–46)
	2-Methyl-1-propanol	78-83-1	1096	1092–1114	43 (41–42)
	1-Butanol	71-36-3	1143	1116–1166	
	2-Methyl-1-butanol	1565-80-6	1200	-	57 (41–56)
	1-Hexanol	111-27-3	1341	1339–1396	
	2-(2-Ethoxyethoxy)-ethanol	111-90-0	1628	1615–1619	45 (59–72)
aldehydes	Hexanal	66-25-1	1077	1048–1120	44 (41–56)
terpenes (2)	D-Limonene	5989-27-5	1186	1176–1238	68 (67–93)
	α-Farnesene	502-61-4	1747	1720–1764	93 (41–69)
benzenic derivatives	Estragole	140-67-0	1724	1624–1661	148 (117–147)

**Table 2:** Semi-quantitative concentrations (nL L^−1^) of VOCs, emitted by intact, freshly cut (cut) and variously treated (C: control with chemical prevention; 55: 55 °C sHWT without chemical prevention; 55_cp: 55 °C sHWT with chemical prevention; 65: 65 °C sHWT without chemical prevention; 65_cp: 65 °C sHWT with chemical prevention) fresh-cut apple slices on days 5, 10 and 13 of storage in sugar syrup at 4 °C. VOCs concentrations given resulted from the emissions of 20 apple slices hermetically enclosed in a 1 L-glass jar at 4 °C for 1 h. Given are means (*n* = 3). Different letters indicate significant differences between means (*p* < 0.05). The different colors help to indicate increasing (green) or decreasing (red) emissions of VOCs compared to intact apples (yellow).

VOC	Time	Initial	Time	C	55	55_cp	65	65_cp
Cumulative VOC concentration	intact	**87 a**	5 d	**445 b–d**	**515 c–e**	**388 b**	**415 b–c**	**425 b–c**
	cut	**618 e**	10 d	**364 b**	**493 c–e**	**320 b**	**379 b**	**354 b**
			13 d	**385 b**	**502 d–e**	**305 b**	**317 b**	**369 b**
Ethyl acetate	intact	**0.86 a**	5 d	**109 c–d**	**152 e**	**80.9 b–c**	**93.3 b–d**	**83.5 b–c**
es1	cut	**1.98 a**	10 d	**104 b–d**	**180 f**	**75.8 b**	**121 d**	**83.2 b–c**
			13 d	**121 d**	**196 f**	**82 b–c**	**106 b–d**	**105 b–d**
Propyl acetate	intact	**nd**	5 d	**3.79 g**	**2.53 d–e**	**2.45 c–e**	**1.78 b–c**	**2.59 d–e**
es2	cut	**2.91 e–f**	10 d	**3.35 f–g**	**2.27 b–e**	**2.31 b–e**	**1.71 b**	**2.39 b–e**
			13 d	**3.74 g**	**2.21 b–e**	**2.11 b–d**	**0.39 a**	**nd**
Butyl acetate *	intact	**2.53 a**	5 d	**27.6 b**	**25.3 b**	**24.4 b**	**26.3 b**	**24.7 b**
es3	cut	**56.0 c**	10 d	**20.0 b**	**18.7 b**	**18.4 b**	**17.7 b**	**17.8 b**
			13 d	**19.0 b**	**17.3 b**	**16.9 b**	**14.9 b**	**17.2 b**
Isobutyl acetate	intact	**1.52 a**	5 d	**21.9 b**	**18.1 b**	**17.9 b**	**22.2 b**	**17.9 b**
es4	cut	**64.1 c**	10 d	**16.7 a–b**	**15.1 a–b**	**13.7 a–b**	**16.1 a–b**	**13.7 a–b**
			13 d	**15.9 a–b**	**15.8 a–b**	**12.3 a–b**	**13.9 a–b**	**14.2 a–b**
2-methylbutyl acetate *	intact	**25.2 a**	5 d	**139 d**	**127 c–d**	**110 b–d**	**114 b–d**	**128 c–d**
es5	cut	**323 e**	10 d	**94.1 b–d**	**94.7 b–d**	**79.5 a–c**	**74.8 a–c**	**91.1 b–d**
			13 d	**87.3 b–d**	**89.9 b–d**	**66.5 a–b**	**62 a–b**	**87.8 b–d**
Pentyl acetate *	intact	**0.78 a**	5 d	**4.36 b**	**3.34 a–b**	**3.36 a–b**	**4.04 b**	**3.16 a–b**
es6	cut	**13.1 c**	10 d	**2.64 a–b**	**2.44 a–b**	**2.02 a–b**	**2.37 a–b**	**1.91 a–b**
			13 d	**2.38 a–b**	**1.83 a–b**	**1.61 a–b**	**1.80 a–b**	**1.43 a–b**
Hexyl acetate *	intact	**3.15 a**	5 d	**5.98 a**	**4.64 a**	**4.45 a**	**5.19 a**	**3.73 a**
es7	cut	**59.8 b**	10 d	**3.48 a**	**3.56 a**	**2.31 a**	**2.85 a**	**2.14 a**
			13 d	**3.15 a**	**2.56 a**	**1.73 a**	**2.13 a**	**1.88 a**
2-Hexen-1-yl acetate	intact	**nd**	5 d	**nd**	**nd**	**nd**	**nd**	**nd**
es8	cut	**22.9 b**	10 d	**nd**	**nd**	**nd**	**nd**	**nd**
			13 d	**nd**	**nd**	**nd**	**nd**	**nd**
Ethyl propionate	intact	**1.46 b–c**	5 d	**3.17 e**	**3.16 e**	**2.03 c–d**	**1.22 b**	**1.65 b–d**
es9	cut	**nd**	10 d	**3.27 e**	**4.23 f**	**2.34 d**	**1.63 b–d**	**1.79 b–d**
			13 d	**5.01 g**	**4.49 f–g**	**2.33 d**	**1.34 b–c**	**1.82 b–d**
Ethyl 2-methylpropanoate	intact	**nd**	5 d	**1.06 b–c**	**2.88 e**	**1.32 b–c**	**1.64 c–d**	**1.24 b–c**
es10	cut	**nd**	10 d	**0.97 b–c**	**3.94 f**	**1.18 b–c**	**2.20 d–e**	**1.31 b–c**
			13 d	**1.35 b–c**	**4.76 g**	**0.63 a–b**	**0.82 a–c**	**0.89 a–c**
Methyl butyrate	intact	**0.63 a**	5 d	**1.37 a–b**	**1.06 a–b**	**1.39 a–b**	**0.77 a–b**	**1.79 b**
es11	cut	**4.20 c**	10 d	**1.13 a–b**	**0.93 a–b**	**1.36 a–b**	**0.63 a**	**1.48 a–b**
			13 d	**1.06 a–b**	**0.93 a–b**	**1.14 a–b**	**0.57 a**	**1.5 a–b**
Methyl 2-methylbutyrate *	intact	**nd**	5 d	**2.27 c–e**	**1.60 b–c**	**2.78 d–g**	**1.09 b**	**2.90 e–g**
es12	cut	**nd**	10 d	**1.97 b–d**	**1.62 b–c**	**3.48 g**	**1.09 b**	**3.27 f–g**
			13 d	**2.44 c–f**	**1.80 b–c**	**2.92 e–g**	**1.06 b**	**2.94 e–g**
Ethyl butyrate *	intact	**nd**	5 d	**12.1 b–c**	**16.8 c–d**	**12.4 b–c**	**13.4 b–c**	**12.5 b–c**
es13	cut	**1.64 a**	10 d	**10.9 b**	**18.4 d**	**11.9 b**	**13.3 b–c**	**11.6 b**
			13 d	**12.9 b–c**	**18.2 d**	**11.0 b**	**10.2 b**	**12.3 b–c**
Ethyl 2-butenoate	intact	**nd**	5 d	**0.54 b–d**	**0.62 b–e**	**0.51 b–c**	**0.91 e–f**	**0.93 e–f**
es14	cut	**nd**	10 d	**0.70 b–f**	**0.82 c–f**	**0.55 b–d**	**0.98 f**	**0.78 b–f**
			13 d	**0.84 d–f**	**0.98 f**	**0.49 b**	**0.79 b–f**	**nd**
Ethyl 2-methylbutanoate *	intact	**nd**	5 d	**25.1 b–c**	**37.9 d–e**	**30.4 b–d**	**23.3 b**	**31.9 b–d**
es15	cut	**0.98 a**	10 d	**25.1 b–c**	**43.3 e–f**	**29.6 b–d**	**33.6 c–d**	**32.8 b–d**
			13 d	**33.7 c–d**	**51.0 f**	**27.4 b–c**	**24.8 b–c**	**33.4 c–d**
Hexyl butyrate *	intact	**5.24 a**	5 d	**1.55 b–d**	**1.92 b–d**	**2.45 b–c**	**2.66 b**	**2.51 b–c**
es16	cut	**5.39 a**	10 d	**1.00 d–e**	**1.16 c–e**	**1.44 b–e**	**1.38 b–e**	**1.65 b–d**
			13 d	**0.63 d–e**	**nd**	**nd**	**nd**	**1.28 c–e**
Hexyl 2-methylbutanoate *	intact	**5.56 a**	5 d	**11 b–e**	**12.9 e**	**12.6 d–e**	**11.3 c–e**	**11.2 c–e**
es17	cut	**8.20 b**	10 d	**11.2 c–e**	**11.1 b–e**	**10.2 b–e**	**9.76 b–d**	**12.3 d–e**
			13 d	**11.4 c–e**	**9.96 b–d**	**9.80 b–d**	**8.67 b–c**	**12.1 d–e**
Ethyl valerate	intact	**nd**	5 d	**0.37 d–e**	**0.47 f–g**	**nd**	**0.39 e–f**	**nd**
es18	cut	**nd**	10 d	**0.34 c–e**	**0.54 g**	**0.26 b–c**	**0.28 b–d**	**nd**
			13 d	**0.38 e**	**0.48 g**	**0.21 b**	**nd**	**0.3 b–e**
Methyl hexanoate	intact	**nd**	5 d	**0.58 b–d**	**1.80 e–f**	**0.54 a–d**	**1.44 e**	**1.01 d**
es19	cut	**2.09 f**	10 d	**0.41 a–b**	**1.89 e–f**	**0.39 a–b**	**0.98 c–d**	**0.44 a–b**
			13 d	**0.49 a–c**	**2.17 f**	**nd**	**nd**	**nd**
Ethyl hexanoate	intact	**nd**	5 d	**13.3 i–j**	**15.7 j**	**9.95 g–h**	**8.04 f–g**	**7.67 e–g**
es20	cut	**1.56 a–b**	10 d	**7.15 d–g**	**11.6 h–i**	**4.88 c–e**	**4.74 b–e**	**4.28 b–d**
			13 d	**6.34 d–f**	**7.72 e–g**	**2.86 a–c**	**2.74 a–c**	**3.22 a–c**
Hexyl hexanoate *	intact	**2.11 a–b**	5 d	**0.98 d–g**	**1.28 c–g**	**1.57 b–e**	**2.48 a**	**1.75 a–d**
es21	cut	**1.92 a–c**	10 d	**0.71 f–g**	**1.07 d–g**	**0.82 e–g**	**1.27 c–g**	**1.43 b–f**
			13 d	**0.56 g**	**0.71 f–g**	**0.73 f–g**	**0.92 e–g**	**1.14 c–g**
Ethanol	intact	**9.72 b**	5 d	**13.1 c**	**27.7 f**	**14.9 c**	**22.0 d–e**	**20.5 d**
al1	cut	**2.21 a**	10 d	**13.1 c**	**27.8 f**	**14.0 c**	**20.9 d–e**	**15.6 c**
			13 d	**15.0 c**	**32.4 g**	**19.7 d**	**23.8 e**	**20.1 d**
2-(2-Ethoxyethoxy)ethanol	intact	**0.65 a**	5 d	**0.35 b–d**	**0.45 b**	**0.43 b**	**0.37 b–d**	**0.38 b–c**
al2	cut	**0.62 a**	10 d	**0.30 c–e**	**0.25 d–e**	**0.22 e**	**0.29 c–e**	**0.22 e**
			13 d	**0.26 c–e**	**0.25 d–e**	**0.28 c–e**	**0.27 c–e**	**0.29 c–e**
2-Methyl-1-propanol	intact	**0.86 a**	5 d	**1.02 a–b**	**0.96 a**	**0.94 a**	**1.21 a–c**	**1.13 a–b**
al3	cut	**2.99 e**	10 d	**0.92 a**	**1.01 a–b**	**0.88 a**	**1.07 a–b**	**0.93 a**
			13 d	**1.07 a–b**	**1.68 d**	**1.50 c–d**	**1.36 b–d**	**1.09 a–b**
1-Butanol *	intact	**1.51 a**	5 d	**1.56 a**	**2.01 b–c**	**1.72 a–b**	**2.13 c**	**1.99 b–c**
al4	cut	**2.82 d**	10 d	**1.59 a**	**1.82 a–c**	**1.72 a–b**	**1.79 a–c**	**1.66 a–b**
			13 d	**1.46 a**	**1.67 a–b**	**1.67 a–b**	**1.67 a–b**	**1.64 a–b**
2-Methyl-1-butanol	intact	**3.24 a**	5 d	**4.25 a–b**	**5.32 b–d**	**4.44 a–c**	**5.14 b–d**	**5.89 c–d**
al5	cut	**9.02 e**	10 d	**4.63 a–c**	**5.58 b–d**	**4.67 b–c**	**4.60 a–c**	**5.21 b–d**
			13 d	**4.51 a–c**	**6.3 d**	**4.77 b–c**	**4.67 b–c**	**5.55 b–d**
1-Hexanol *	intact	**1.44 a**	5 d	**2.90 b**	**3.99 b–c**	**3.07 b**	**4.54 c**	**3.60 b–c**
al6	cut	**3.94 b–c**	10 d	**3.06 b**	**4.21 b–c**	**3.08 b**	**3.39 b–c**	**2.84 b**
			13 d	**2.88 b**	**3.93 b–c**	**3.27 b–c**	**3.52 b–c**	**3.11 b**
2-Butanone	intact	**2.67 a–e**	5 d	**0.86 a–b**	**4.71 e–f**	**3.62 c–e**	**8.89 g**	**6.75 f–g**
k1	cut	**0.33 a**	10 d	**0.73 a–b**	**7.32 g**	**1.96 a–d**	**4.68 e–f**	**4.22 d–e**
			13 d	**1.32 a–c**	**7.12 g**	**2.44 a–e**	**3.30 b–e**	**3.76 c–e**
1-Penten-3-one	intact	**nd**	5 d	**1.35 b**	**2.29 b–d**	**1.77 b–c**	**3.35 e–f**	**1.98 b–c**
k2	cut	**nd**	10 d	**3.71 e–f**	**3.98 f**	**3.21 d–f**	**5.16 g**	**3.72 e–f**
			13 d	**3.52 e–f**	**2.78 c–e**	**4.18 f**	**4.06 f**	**3.76 e–f**
Hexanal *	intact	**0.50 a**	5 d	**3.23 b–d**	**6.47 f–g**	**4.66 d–f**	**7.14 g**	**5.25 d–g**
ad1	cut	**1.92 a–b**	10 d	**4.49 d–f**	**5.08 d–f**	**3.72 b–d**	**6.26 e–g**	**4.43 d–e**
			13 d	**4.19 c–d**	**2.30 a–c**	**3.46 b–d**	**3.77 b–d**	**3.81 b–d**
Estragole	intact	**0.30 b–e**	5 d	**0.61 g**	**0.42 c–f**	**0.45 d–g**	**0.35 b–f**	**0.47 e–g**
bd1	cut	**0.41 b–f**	10 d	**0.52 f–g**	**nd**	**nd**	**0.29 b–d**	**0.37 b–f**
			13 d	**0.51 f–g**	**nd**	**0.26 b–c**	**0.24 b**	**0.36 b–f**
D-Limonene	intact	**3.27 a**	5 d	**6.26 a–d**	**10.4 e**	**9.77 e**	**7.54 b–e**	**8.45 d–e**
tp1	cut	**4.93 a–c**	10 d	**4.74 a–b**	**4.54 a**	**9.54 e**	**7.65 c–e**	**8.67 d–e**
			13 d	**4.70 a–b**	**4.40 a**	**9.06 d–e**	**5.23 a–c**	**9.28 e**
α-Farnesene	intact	**13.8 a–c**	5 d	**24.0 d–e**	**18.8 a–d**	**21.3 c–e**	**16.8 a–d**	**28.0 e**
tp2	cut	**19.1 a–d**	10 d	**17.4 a–d**	**14.4 a–c**	**14.9 a–c**	**15.0 a–c**	**20.5 b–e**
			13 d	**16.5 a–d**	**10.6 a**	**12.1 a–b**	**12.5 a–b**	**17.7 a–d**

* marked VOCs described as generally important “character impact” compounds in apples [29]. nd = not detected/below detection limit; dark green = ≥500% increase; light green = ≥33% increase; yellow = ≤33% increase/decrease; light red = ≥80% decrease; dark red = ≥500% decrease.

**Table 3:** Treatment-specific qualitative changes in the emissions of relevant VOCs and corresponding aroma threshold values of fresh-cut apple slices stored in sugar syrup at 4 °C for up to 13 days.

Treatment	Reaction	VOCs	Aroma Threshold Values (nL L^−1^)
all sHWT	↑	Ethanol	8–900 ^a^/>1 × 10^5,b^
	↓	Propyl acetate	2000–11,000 ^a,b^
		Estragole	n/a
sHWT without cp	↑	Ethanol	8–900 ^a^/>1 × 10^5,b^
sHWT combined with cp	↑	D-limonene	4–229 ^a^
	↓	Ethyl 2-butenoate	n/a
only 55 °C sHWT	↑	2-methyl-1-propanol	360–3300 ^a^
only 55 °C sHWT without cp	↑	Ethyl acetate	5–13,500 ^b^
		Ethyl 2-methylpropanoate	0.01–1 ^a^
		Ethyl butyrate *	0.1–18 ^a^
		Ethyl 2-methylbutanoate *	0.006–0.1 ^a,b^
		Ethyl valerate	1.5–5 ^a,b^
		Methyl hexanoate	10–87 ^a^
		2-butanone	n/a
	↓	Estragole	n/a
		1-penten-3-one	400 ^a^
only 55 °C sHWT combined with cp	↓	Ethyl acetate	5–13,500 ^a,b^
only 65 °C sHWT	↓	Propyl acetate	2000–11,000 ^a,b^
only sHWT at 65 °C or combined with cp	↓	Ethyl propionate	9–45 ^a^
		Ethyl hexanoate	0.3–5 ^a^
		Methyl hexanoate	10–87 ^a^

sHWT: short-term hot water treatment; cp: chemical prevention by applying organic acid dipping of fresh-cut slices; 55 °C/65 °C: sHWT at the specified temperature; green ↑: increase; red ↓: decrease. Given are aroma threshold values according to ^a^ Burdock [30] and ^b^ Dixon and Hewett [29]. * marked VOCs described as generally important “character impact” compounds in apples [29].
